# Characteristics of a hepatitis C patient cohort at a specialized tertiary care facility: Identifying criteria to improve the allocation of public health resources

**DOI:** 10.6061/clinics/2019/e1286

**Published:** 2019-10-23

**Authors:** Maria Laura Mariano de Matos, Rosário Quiroga Ferrufino, Ana Catharina de Seixas Santos Nastri, Fatuma Catherine Atieno Odongo, Aléia Faustina Campos, André Machado Luiz, Gaspar Lisboa-Neto, Steven S. Witkin, Maria Cássia Mendes-Correa

**Affiliations:** I Departamento de Molestias Infecciosas e Parasitarias, Faculdade de Medicina FMUSP, Universidade de Sao Paulo, SP, BR; II Department of Obstetrics and Gynecology, Weill Cornell Medicine, New York, New York (S.S.W.), USA; III Laboratorio de Virologia – LIM 52, Instituto de Medicina Tropical (IMT), Sao Paulo, SP, BR

**Keywords:** Hepatitis C, Complexity, Tertiary Care, Public Health, Brazil

## Abstract

**OBJECTIVES:**

Our objective was to analyze, in a population treated for hepatitis C infection at a tertiary care treatment unit, the prevalence of comorbidities and extrahepatic manifestations, the range and degree of the clinical complexity and the associations between advanced liver disease and clinical variables.

**METHODS:**

Medical records from chronically infected hepatitis C patients seen at a dedicated treatment facility for complex cases in the Infectious Diseases Division of Hospital das Clínicas in Brazil were analyzed. Clinical complexity was defined as the presence of one or more of the following conditions: advanced liver disease (Metavir score F3 or F4 and/or clinical manifestations or ultrasound/endoscopy findings consistent with cirrhosis) or hepatocellular carcinoma and/or 3 or more extrahepatic manifestations and/or comorbidities concomitantly.

**RESULTS:**

Among the 1574 patients analyzed, only 41% met the definition of being clinically complex. Cirrhosis or hepatocarcinoma was identified in 22.2% and 1.8% of patients, respectively. According to multiple logistic regression analysis, male sex (*p*=0.007), age>40 years (*p*<0.001) and the presence of metabolic syndrome (*p*=0.008) were independently associated with advanced liver disease.

**CONCLUSION:**

The majority of patients did not meet the criteria for admittance to this specialized tertiary service, reinforcing the need to reevaluate public health policies. Enhanced utilization of existing basic and intermediate complexity units for the management of less complex hepatitis C cases could improve care and lower costs.

## INTRODUCTION

Designing services with the capacity and expertise to optimally allocate resources to meet the needs of hepatitis C-infected populations requires a detailed understanding of their characteristics and healthcare needs. In 2016, the World Health Organization (WHO) adopted viral hepatitis elimination targets, aiming for a 90% reduction in new infections and a 65% reduction in mortality within the next 14 years (1). Brazil, with an estimated 670,000 hepatitis C-infected individuals (2), is one of nine countries currently on track to achieve hepatitis C elimination by 2030 (3). In 2015, the Ministry of Health concluded price negotiations with pharmaceutical companies and gained access to affordable direct-action antivirals (4). As of 2018, the Ministry of Health is committed to providing free treatment for hepatitis C to all those infected (5).

Information derived from a reference center at a tertiary care hospital can serve as a guide to identify specific deficiencies, revise strategies to better meet the needs of infected individuals and more effectively pinpoint areas for improvements in public health interventions. The aims of this study are to describe, in a large patient population treated at a hepatitis C tertiary medical care unit in Brazil, (1) their demographic and clinical characteristics, (2) prevalence of comorbidities or extrahepatic manifestations, (3) the range and degree of clinical complexity in this population, and (4) the associations between advanced liver disease and clinical variables. Based on this information, new recommendations for improved clinical public health services are described.

## MATERIAL AND METHODS

### Patient population

In this retrospective study, the patients analyzed were those registered at the Hepatitis C Outpatient Clinic in the Infectious Diseases Division of Hospital das Clinicas, a tertiary care hospital in Sao Paulo, Brazil. This is an ambulatory reference center specifically for patients with complex complications of hepatitis C viral infections.

Hepatitis C-associated comorbidities were defined as any cardiovascular, renal, neurological, psychiatric, dermatological, rheumatologic or endocrinological condition associated with this infection that required clinical or pharmacological intervention from the attending physician. Additional comorbidities included conditions (gastrointestinal, pulmonary, infectious, musculoskeletal, otorhinolaryngological, urologic and alcohol consumption) not traditionally associated with HCV that could have a negative impact on the response to hepatitis C treatment or even postpone or contraindicate intervention.

Clinical complexity was thus defined as the presence of advanced liver disease (Metavir score F3/F4 and/or clinical manifestations or ultrasound/endoscopy findings consistent with cirrhosis) or hepatocarcinoma and/or 3 or more comorbidities or extrahepatic manifestations, including cryoglobulinemia, vasculitis and other autoimmune phenomena, lymphoproliferative disorders, systemic scleroderma, porphyria cutanea tarda, rheumatoid-like arthritis, lichen planus or metabolic syndrome. Metabolic syndrome was defined as proposed by the National Cholesterol Education Program (NCEP) Adult Treatment Panel III (ATP III) (6), that is as the presence of any three of the following five traits: (1) abdominal obesity, defined as a waist circumference ≥102 cm (40 in) in men and ≥88 cm (35 in) in women; (2) serum triglycerides ≥150 mg/dL (1.7 mmol/L) or drug treatment for elevated triglycerides; (3) serum high-density lipoprotein (HDL) cholesterol <40 mg/dL (1 mmol/L) in men and <50 mg/dL (1.3 mmol/L) in women or drug treatment for low HDL cholesterol; (4) blood pressure ≥130/85 mmHg or drug treatment for elevated blood pressure; or (5) fasting glucose ≥100 mg/dL (5.6 mmol/L) or drug treatment for elevated blood glucose.

The included patients were hepatitis C-infected patients treated from January 2014 to December 2016, age over 18 years and viremic at enrolment. The excluded patients were those who were negative for hepatitis C RNA (spontaneous virus clearance) and those coinfected with HIV. Outpatient care of HIV-infected patients occurred at another outpatient unit, a specific ambulatory setting specifically for HIV-infected patients and their sexual partners.

### Data collection

Clinical and laboratory data were extracted from a database routinely prepared and maintained for each patient. The variables analyzed included age, sex, HIV status, hepatitis A and B or HTLV coinfection, hepatitis C virus genotype, stage of liver fibrosis, and the presence of extrahepatic manifestations and comorbidities. All patients had undergone a percutaneous liver biopsy, except those with clinical manifestations or ultrasound/endoscopy findings consistent with cirrhosis. On histopathological analysis, structural liver injury was assessed and graded according to the Metavir classification system (7). Extrahepatic manifestations of hepatitis C infection were diagnosed by standard protocols in the appropriate clinical laboratory.

This study was approved by the Ethics Committee for the Analysis of Research Projects (Comissão de Ética para Análise de Projetos de Pesquisa-CAPPesq) of the Clinics Hospital of the Medical School of the University of São Paulo (Hospital das Clínicas da Faculdade de Medicina da Universidade de São Paulo - HC-FMUSP) under protocol no. CAAE: 37392414.5.0000.0068, and all subjects provided informed written consent.

### Statistical analysis

We initially conducted an analysis of the frequency distribution for each selected variable. Pearson’s chi-square test was used to compare proportions of demographic, virological, and clinical variables among patients characterized as complex or not complex and among individuals with or without advanced liver disease. Continuous parameters were compared between groups using Student’s t-test. Odds ratios (ORs) were calculated as association measures, with their respective 95% confidence intervals (95% CI). Two-tailed *p-*values were calculated and considered statistically significant if <0.05. Variables with p values less than 0.20 on bivariate analysis were selected for multivariate analysis.

Multiple logistic regression analysis was performed to examine the association of variables with advanced liver disease. Statistical analyses were performed using Stata software version 13.0 (Stata Corp., College Station, TX, USA).

## RESULTS

A total of 2,194 patients were identified in the database, and 1574 of them were included in the final study. The remaining 620 patients were excluded due to testing negative for hepatitis C RNA (n=112), having HIV coinfection (n=452) or lacking complete clinical data (n=56).

Table 1 summarizes the demographic and clinical characteristics of the study population. Their mean age (standard deviation) was 54.8 (12.3) years, and a small majority were female (52.6%). HCV genotype 1 was most prevalent (75.4%), followed by genotypes 3 (19.8%), 2 (3.9%), 4 (0.4%) and 5 (0.4%). All patients underwent a liver fibrosis evaluation: 12.6% had severe fibrosis (F3), 25.2% had moderate fibrosis (F2), 33.7% had mild fibrosis (F1) and 6.4% had no evidence of fibrosis. In addition, 22.2% had cirrhosis. Hepatocarcinoma was diagnosed in 1.8% of the patients. The majority of patients (62.3%) had at least one HCV-associated comorbidity, while 41.2% had another comorbidity not typically regarded as being HCV-associated.

Among our study population, only 646 (41%) met the definition of being clinically complex. The associations between clinical complexity and demographic and clinical characteristics are shown in Table 2. The only variable associated with clinical complexity was age above 40 years (*p*<0.001).

Table 3 details the associations between advanced liver disease and other characteristics. According to bivariate analysis, advanced liver disease was independently associated with male sex (*p*=0.012), age over 40 years (*p*<0.001), HCV-associated comorbidities (*p*=0.004), other comorbidities (*p*=0.025) and the presence of metabolic syndrome (*p*=0.001). According to multiple logistic regression analysis, male sex (*p*=0.007), age >40 (*p*<0.001) and the presence of metabolic syndrome (*p*=0.008) remained independently associated with advanced liver disease (Table 4).

## DISCUSSION

The present study describes the demographic and clinical characteristics of patients treated in a hepatitis C tertiary medical care unit in Brazil and the associations between advanced disease and clinical variables. We observed that approximately one-third of subjects had advanced liver disease, and 41% were clinically complex. Our observation that 34.8% of patients had advanced liver disease parallels a prior national investigation reporting that 28.8% of chronic hepatitis C cases in Brazil present with advanced forms of liver disease (2).

In our study, the level of complexity of the patients analyzed took into consideration the presence of comorbidities associated with hepatitis C virus and/or the presence of advanced liver disease and its complications. Following the convention of Feinstein (8), the term comorbidity refers to any distinct clinical entity that has existed or that may occur during the course of hepatitis C disease. As such, these are not limited to those classically associated with this viral infection or its treatment. It is important to mention, however, that the presence of comorbidities may not necessarily significantly impact hepatitis C clinical management. This is also true with respect to hepatitis C-associated extrahepatic manifestations. Numerous extrahepatic manifestations have been reported (9,10) that vary in severity, from clinical situations often unobserved to highly disabling conditions. However, to quantify the clinical situations of greater complexity, we included only comorbidities that resulted in a clinical or pharmacological intervention.

All extrahepatic manifestations were considered in the analysis, as detailed in the inclusion criteria. In addition, we took into account the presence of 3 or more comorbidities or extrahepatic manifestations to define clinical complexity. According to these criteria, only 41% of the population analyzed had clinical comorbidities or had advanced liver disease that justified their stay in a high-complexity hospital unit for the care of patients with hepatitis C.

The association between advanced liver disease and clinical variables was evaluated. According to multivariate analysis, advanced liver disease was independently associated with male sex, age over 40 years and the presence of metabolic syndrome. The association of age over 40 years with advanced liver disease has been extensively described previously and is confirmed by our data (6,11-13). Patients’ age at the time of hepatitis C diagnosis has been shown to be a risk factor for the progression of liver fibrosis, liver cirrhosis, and hepatocellular carcinoma (11-13). Additionally, the association between hepatitis C and metabolic syndrome has been evaluated previously. HCV is thought to induce metabolic alterations resulting in hypolipidemia, hepatic steatosis, insulin resistance (IR), metabolic syndrome, and diabetes. These effects may lead to clinically relevant consequences affecting both liver disease progression and response to antiviral therapy (14-16).

It is important to mention the limitations of this study. The investigation was cross-sectional and retrospective. Variables were obtained from a database used for patient follow-up and, therefore, may not have been complete. In addition, our study presents the findings of a single tertiary center and may possibly not be representative of other tertiary centers in Brazil or elsewhere.

Our study contained the largest cohort of patients with chronic hepatitis C analyzed in a tertiary center in Brazil, and the findings are consistent with and reinforce conclusions from prior studies. The analysis reveals that the majority of patients did not meet the criteria for admittance to this highly complex and specialized tertiary service. Many of these individuals could have been satisfactorily followed in other lower complexity health units. Thus, our data reinforces a continued need to establish more appropriate resource allocation and public health policies to more effectively manage individuals with hepatitis C infection. Health services in Brazil, as in other low- and middle-income countries, have a limited capacity to provide specialized care for all deserving conditions.

There are many competing priorities in health care, and all require adequate resources and attention. The utilization of health care providers at basic and intermediate complexity units for the diagnosis and management of hepatitis C-infected patients who are presently managed in more highly specific services could result in the improved utilization of existing resources at a lower cost. By working in collaboration, these different levels of services could more effectively advance the goals of increasing the availability of treatment and potential for the cure of hepatitis C-infected patients. We believe that the distribution and tasks attributed to each level of care should also be adjusted according to regional demand and availability. The absence of a structured cascade of care may lead to a delay in the achievement of the targets set by the Brazilian Ministry of Health for the elimination of hepatitis C.

## AUTHOR CONTRIBUTIONS

Mendes-Correa MC and Matos MLM were responsible for the study conception and design. Matos MLM, Ferrufino RQ, Nastri ACSS, Odongo FCA, Campos AF and Luiz AM collected the data. Mendes-Correa MC, Lisboa-Neto G and Matos MLM were responsible for analyzing the results. Witkin SS, Matos MLM and Mendes-Correa MC were responsible for critical revision of the manuscript.

## Figures and Tables

**Table 1:** Demographics and clinical characteristics of the study population.

Characteristic	Value
Female sex	52.6%
Mean age (SD)	54.8 (12.3) years
Age >40 years	82.0%
HCV genotype	
1	75.4%
2	3.9%
3	19.8%
4	0.4%
5	0.45
Liver fibrosis	
None (METAVIR F0)	6.4%
Mild (METAVIR F1)	33.7%
Moderate (METAVIR F2)	25.2%
Severe (METAVIR F3)	12.6%
Cirrhosis	22.2%
Extrahepatic manifestations^a^	8.3%
Hepatocarcinoma	1.8%
HCV-associated comorbidity^b^	62.3%
Non-HCV-associated comorbidity^c^	41.2%

A total of 1,574 HCV-infected individuals were evaluated.

a Extrahepatic manifestations include the presence of cryoglobulinemia, vasculitis and other autoimmune phenomena, lymphoproliferative disorders, systemic scleroderma, porphyria cutanea tarda, rheumatoid-like arthritis and lichen planus.

b HCV-associated comorbidity includes any cardiovascular, renal, neurological, psychiatric, dermatological, rheumatologic, endocrinological condition known to be associated with this infection that required clinical or pharmacological intervention from the attending physician.

c Non-HCV-associated comorbidity includes any clinical condition (gastrointestinal, pulmonary, infectious, musculoskeletal, otorhinolaryngological and urologic) not traditionally associated with HCV that could have a negative impact on the response to hepatitis C treatment or even postpone or contraindicate intervention.

**Table 2:** Demographic and clinical characteristics of patients according to the presence or absence of clinical complexity.

	Complexity			
	No	Yes		
Characteristic	N=928	N=646	OR (IC 95%)	*p-*value
Male sex	45.8%	49.7%	1.16 (0.96-1.43)	0.128
Female sex	54.2%	50.3%	NS	
Age ≤40 years	23.0%	11.0%	NS	
>40 years	77.0%	89.0%	2.43 (1.08-3.22)	<0.001
Genotype				
1	77.5%	72.4%		0.131
2	3.8%	4.0%		
3	17.9%	22.6%		
4+5	0.9%	0.9%		
Mean age, years (SD)	52.1 (12.2)	58.8 (11.3)		<0.001

Clinical complexity is defined as the presence of one or more of the following conditions: advanced liver disease (Metavir score F3 or F4 and/or clinical manifestations or ultrasound/endoscopy findings consistent with cirrhosis) or its consequences (hepatocellular carcinoma and/or portal hypertension) and/or 3 or more extrahepatic manifestations and/or comorbidities concomitantly.

**Table 3:** Demographic and clinical characteristics of patients according to the presence or absence of advanced liver disease* according to univariate analysis.

	Complexity			
	No	Yes		
Characteristic	N=1027	N=547	OR (IC 95%)	*p-*value
Male sex	45.1%	51.7%	1.31 (1.06-1.61)	0.012
Female sex	54.9%	48.3%	NS	
Age ≤40 years	22.2%	10.2%	NS	
>40 years	77.8%	89.8%	2.50 (1.82-3.42)	<0.001
Genotype				
1	77.3%	71.8%	NS	
2	3.7%	4.2%	NS	
3	18.1%	23.0%	NS	
4+5	0.9%	0.9%	NS	
HCV-associated comorbidity	59.8%	67.1%	1.37 (1.10-1.70)	0.004
Non-HCV-associated comorbidity	39.1%	45.0%	1.27 (1.03-1.56)	0.025
Metabolic syndrome	8.4%	13.7%	1.73 (1.25-2.41)	0.001
Extrahepatic manifestations	7.8%	9.1%	NS	

Advanced liver disease* is defined as Metavir score F3 or F4 and/or clinical manifestations or ultrasound/endoscopy findings consistent with cirrhosis.

**Table 4:** Factors independently associated with advanced liver disease according to multiple logistic regression analysis.

Characteristic	OR (adjusted)	95% CI	*p* value
Male sex	1.34	1.08-1.65	0.007
Age >40 years	2.43	1.77-3.34	<0.001
Metabolic syndrome	1.57	1.12-2.18	0.008
